# Effects of Undernutrition on Swallowing Function and Activities of Daily Living in Hospitalized Patients: Data from the Japanese Sarcopenic Dysphagia Database

**DOI:** 10.3390/nu15051291

**Published:** 2023-03-06

**Authors:** Sayaka Abe, Yoji Kokura, Keisuke Maeda, Shinta Nishioka, Ryo Momosaki, Hiroki Matsuoka, Yasuomi Tomii, Shinnosuke Sugita, Kenta Shimizu, Nanami Esashi, Hidetaka Wakabayashi

**Affiliations:** 1 Department of Nutrition, Sapporonishimaruyama Hospital, Sapporo 064-0944, Japan; 2 Department of Nutritional Management, Keiju Hatogaoka Integrated Facility for Medical and Long-Term Care, Hosu 927-0023, Japan; 3 Department of Geriatric Medicine, Hospital, National Center for Geriatrics and Gerontology, Obu 474-8511, Japan; 4 Department of Clinical Nutrition and Food Service, Nagasaki Rehabilitation Hospital, Nagasaki 850-0854, Japan; 5 Department of Rehabilitation Medicine, Mie University Graduate School of Medicine, Tsu 514-0001, Japan; 6 Department of Rehabilitation, Sapporonishimaruyama Hospital, Sapporo 064-0944, Japan; 7 Social Welfare Corporation, Tobetsu Town Council of Social Welfare, Ishikari 064-0234, Japan; 8 Department of Rehabilitation Medicine, Tokyo Women’s Medical University Hospital, Tokyo 162-8666, Japan

**Keywords:** nutritional status, nutrition disorders, deglutition disorders, functional status

## Abstract

This retrospective cohort study examined the effects of undernutrition on swallowing function and activities of daily living in hospitalized patients. Data from the Japanese Sarcopenic Dysphagia Database were used, and hospitalized patients aged ≥20 years with dysphagia were included in the analysis. Participants were assigned to the undernutrition or normal nutritional status group based on the Global Leadership Initiative on Malnutrition criteria. The primary outcome was the Food Intake Level Scale change, and the secondary outcome was the Barthel Index change. Among 440 residents, 281 (64%) were classified under the undernutrition group. The undernutrition group had a significantly higher Food Intake Level Scale score at baseline and Food Intake Level Scale change (*p* = 0.001) than the normal nutritional status group. Undernutrition was independently associated with the Food Intake Level Scale change (B = −0.633, 95% confidence interval = −1.099 to −0.167) and the Barthel Index change (B = −8.414, 95% confidence interval = −13.089 to −3.739). This was defined as the period from the date of admission to the hospital until discharge or 3 months later. Overall, our findings indicate that undernutrition is associated with reduced improvement in swallowing function and the ability to perform activities of daily living.

## 1. Introduction

Undernutrition is frequent in patients with dysphagia, which results in worse clinical outcomes. Numerous diseases cause dysphagia including cerebrovascular disease (e.g., stroke), neurodegenerative disease (e.g., amyotrophic lateral sclerosis), dementia and head and neck cancer [1]. The prevalence of dysphagia is 11–33% in community-dwelling older adults [2,3], 53% in institutionalized residents [4] and 24–50% in hospitalized patients [5,6]. There are also reports of a high incidence of dysphagia and undernutrition co-occurrence [5,7,8]. Dysphagia patients who are undernourished have longer hospital stays, higher risk of complications [6] and higher mortality rates [9]. Therefore, these patients should be evaluated for undernutrition.

Sarcopenic dysphagia and presbyphagia have recently received attention as causes of dysphagia. Sarcopenic dysphagia is a swallowing disorder caused by sarcopenia of the entire body as well as the swallowing muscle. Low muscle mass, age, undernutrition, lack of independence in daily living and low tongue pressure have recently been identified as risk factors for sarcopenic dysphagia [1]. Since 1992, undernutrition has been recognized as a risk factor for feeding and swallowing difficulties [10]. Undernutrition can reduce overall muscle mass and strength, resulting in a decrease in swallowing-related muscle tone. Undernutrition is caused by starvation, invasion and cachexia, and the dysphagia caused by undernutrition is classified as sarcopenia dysphagia [1]. Furthermore, the concept of presbyphagia refers to age-related sarcopenia of the lingual and supraspinal muscle groups [11]. Hypophagia is not uncommon in patients with presbyphagia owing to decreased muscle mass and function of swallowing-related muscles.

A few studies have assessed patients with dysphagia using the Global Leadership Initiative on Malnutrition (GLIM) criteria, which is a diagnostic method for evaluating undernutrition proposed by several global societies [12]. The GLIM criteria involve the assessment of muscle mass, body mass index and disease effects. Hence, it can be used for the nutritional assessment of adults with dysphagia [13]. However, a survey on nutritional assessment methods among patients admitted to internal medicine departments has found that only 20% of studies used the GLIM criteria [14]. In previous studies, 90% of patients with dysphagia who were admitted to a rehabilitation hospital presented with undernutrition diagnosed using the GLIM criteria [15,16]. In addition, severe undernutrition according to the same criteria was found to be associated with poor improvement in dysphagia [15]. However, previous studies only included patients with sarcopenic dysphagia. Therefore, whether undernutrition diagnosed using the GLIM criteria is a useful prognostic indicator of swallowing and physical function in patients with dysphagia should be examined.

The current study aimed to examine the effects of undernutrition diagnosed by the GLIM criteria on activities of daily living and swallowing function in hospitalized patients with dysphagia.

## 2. Materials and Methods

### 2.1. Study Design and Participants

This was a retrospective cohort study. Patients from the Japanese Sarcopenia Dysphagia Database, which was jointly established by the Rehabilitation Nutrition Database Committee of the Japanese Association of Rehabilitation Nutrition and the Japanese Working Group on Sarcopenic Dysphagia, were enrolled in this study [17]. Although whole-body sarcopenia and malnutrition are known risk factors for the development of sarcopenic dysphagia, other risk factors and associated factors remain unknown. Therefore, we created the Japanese Sarcopenic Dysphagia Database, which can be used by many hospitals to collect more information on sarcopenic dysphagia. The Japanese Association of Rehabilitation Nutrition and the Japanese Working Group on Sarcopenic Dysphagia recruited participating facilities. To ensure data accuracy, the REDCap data input manual was distributed to all data entry personnel at the participating facilities. The timing of baseline data entry was at admission. At the baseline and follow-up, entry personnel recorded the patients’ data. The timing of outcome measurement was at discharge or 3 months later. At the follow-up visit, the outcome was classified as discharge to home, transfer to other hospitals/facilities, continuation of hospitalization or home stay or death. Nineteen hospitals, including nine acute-care, eight rehabilitation and two long-term care hospitals, and one home-visit rehabilitation team were registered in this database. The registration period was from April 2018 to March 2021. The inclusion criteria were patients aged >20 years who presented with dysphagia, which was defined as a Food Intake Level Scale [18] score of ≤8. Food Intake Level Scale scores of 1–3 relate to various degrees of nonoral feeding; 4–6 to various degrees of oral food intake and alternative nutrition; 7 and 8 to various degrees of oral food intake alone; 9 to no dietary restriction but medical consideration given and 10 indicates normal oral food intake. The exclusion criteria were patients with missing data. This study was approved by the Ethics Committee of Sapporo Nishi Maruyama Hospital, Keijinkai (1 November 2019). All patients provided informed consent prior to enrolment in the database.

### 2.2. Data Collection

The following data were included in the database registration: age, sex, body mass index, calf circumference, handgrip strength, C-reactive protein level, serum albumin levels and Charlson Comorbidity Index [19]. Body mass index was calculated by dividing body weight (kg) by height squared (m^2^). Charlson Comorbidity Index was used to assess comorbidities, with higher scores indicating greater comorbidity severity and risk of mortality. At baseline and follow-up, the Food Intake Level Scale and Barthel Index scores were calculated. The Barthel Index includes 10 items as follows: feeding, moving from a wheelchair to bed and back, grooming, transferring to and from a toilet, bathing, walking on a level surface, going up and down stairs, dressing, bowel continence and bladder continence. The degrees of functional ability for each Barthel Index category vary, with higher scores indicating complete independence and lower scores indicating less physical function. Clinical, rehabilitation-related and nutrition-related data were entered based on the judgement of physicians, rehabilitation-related professionals and ward staff and dieticians, respectively. Registry guidelines were developed for measurement.

### 2.3. Nutritional Assessment

In this study, nutritional status was diagnosed using the GLIM criteria [20]. The GLIM criteria were developed to standardize the diagnosis of adult undernutrition in clinical practice worldwide. The steps involved in assessments using the GLIM criteria are as follows: The first step is to screen the candidates using a validated screening tool. The Mini Nutritional Assessment Short-Form was used as the screening tool in this study [21]. If the screening indicates that the patient is at risk of malnutrition, the patient moves on to the next step. The second step is to assess each of the three phenotypic and two etiologic criteria. The phenotypic criteria comprise the following three components: unintentional weight loss, low body mass index and loss of muscle mass. The phenotypic criteria included weight loss (≥5% within the last 6 months and ≥10% after >6 months), low body mass index (18.5 kg/m^2^ in patients aged <70 years and <20.0 kg/m^2^ in those aged ≥70 years) and reduced muscle mass [22,23]. There are two etiologic criteria: decreased food intake or digestive/absorptive capacity and disease burden/inflammatory involvement. The etiologic criteria used in this study were reduced food intake (1 week or any reduction for >2 weeks), assimilation problems (e.g., dysphagia, vomiting and diarrhea) and disease burden/inflammatory condition (e.g., congestive heart failure, chronic obstructive pulmonary disease, chronic kidney disease and cancer). When one or more of the presenting phenotypic and etiologic criteria apply, undernutrition is diagnosed.

### 2.4. Outcome

The primary outcome was improvement in the Food Intake Level Scale. The Food Intake Level Scale is a 10-point scale for assessing dysphagia [18]. Food Intake Level Scale scores of 1–3 indicated parenteral intake; 4–6 indicated oral food intake and alternative nutrition; 7 and 8 indicated oral food intake alone; 9 indicated no dietary restrictions but with medical considerations and 10 indicated no issues with swallowing. The Food Intake Level Scale change was calculated by subtracting the Food Intake Level Scale score at baseline from that at follow-up. A larger increase in Food Intake Level Scale change indicated a greater improvement in dysphagia. The secondary outcome was the Barthel Index change, a widely used measure of activities of daily living [24]. The Barthel Index comprises 10 items: bathing, grooming, eating, dressing, defecation, bladder, toilet movement, stairs, transferring and moving. Bathing and grooming were rated on a scale of 0 or 5. Eating, dressing, defecation, bladder, toilet movement and stairs were rated on a scale of 0, 5 or 10. Transferring and moving were rated using a four-point scale (0, 5, 10 or 15). The highest Barthel Index score was 100, with higher scores indicating a better ability to perform activities of daily living. The Barthel Index gain was calculated by subtracting the Barthel Index score at baseline from that at follow-up. A larger increase in Barthel Index change indicated a greater improvement in performing activities of daily living [25].

### 2.5. Statistical Analyses

Data analysis was performed using the Statistical Package for the Social Sciences software version 26 (IBM; Armonk, New York, the USA). Parametric data are expressed as mean ± standard deviation and nonparametric data as median and interquartile range. Categorical data are presented as *n* (%). The Student’s t-test, Mann–Whitney U test, chi-square test and Fisher’s exact test were used to analyze differences between the undernutrition and normal nutritional status groups. The Food Intake Level Scale gain and Barthel Index gain were compared between the undernutrition and normal nutritional status groups in the univariate analysis. In addition, multiple regression analysis was performed with the Food Intake Level Scale gain and Barthel Index gain as the objective variables and undernutrition as the explanatory variable. The covariates were age, sex, Charlson Comorbidity Index and setting at the time of the survey. A *p*-value of <0.05 was considered statistically significant. The follow-up was performed at discharge or 3 months after admission, at which time changes were calculated.

## 3. Results

**Figure 1:**
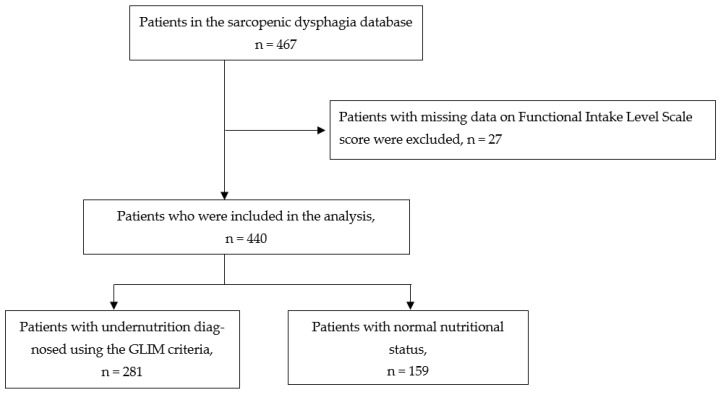
A flowchart of participant selection.

The total number of registered patients in the Japanese Sarcopenic Dysphagia Database was 467. Among them, 27 patients with missing data were excluded. Finally, 440 registered patients were included in the analysis (Figure 1). Rehabilitation hospitals were the most common prehospitalization facilities (47.5%), followed by acute-care hospitals (42.5%), long-term care hospitals (9.3%) and others (0.7%).

Table 1 displays the demographic characteristics of the participants at baseline. The mean age of the patients was 80.2 ± 10.9 years. Furthermore, 281 (64%) patients were classified as undernourished according to the GLIM criteria and were included in the undernutrition group. Compared with the normal nutritional status group, the undernutrition group was significantly older and had a high C-reactive protein level and Charlson Comorbidity Index (*p* < 0.001). The undernutrition group had a significantly lower serum albumin level, body mass index, calf circumference and handgrip strength than the normal nutritional status group (*p* < 0.001).

Table 2 presents the results of analysis using the GLIM criteria for the undernutrition and normal nutritional status groups. The results of the phenotypic criteria are given below. The percentage of unintentional weight loss was significantly higher in the undernutrition group (41 (9.4%)) than in the normal nutritional status group (7 (1.6%); *p* < 0.001). The percentage of low body mass index was significantly higher in the undernutrition group (158 (36.0%)) than in the normal nutrition status group (53 (12.1%); *p* < 0.001). The percentage of decreased muscle mass was significantly greater in the undernutrition group (276 (63.0%)) than in the normal nutritional status group (118 (26.9%); *p* < 0.001). In terms of etiological criteria, the percentage of decreased food intake or assimilation was significantly higher in the undernutrition group (160 (36.4%)) than in the normal nutritional status group (8 (1.8%); *p* < 0.001). The rate of disease burden/inflammation was significantly higher in the undernutrition group (170 (38.7%)) than in the normal nutrition status group (13 (3.0%); *p* < 0.001).

Table 3 displays the results of univariate analysis of Food Intake Level Scale and Barthel Index for the undernutrition and normal nutrition status groups. The median Food Intake Level Scale score at baseline was significantly higher (*p* < 0.001) in the undernutrition group (median, 7; interquartile range, 5–8) than in the normal nutrition status group (median, 7; interquartile range, 2–7). At follow-up, the median Food Intake Level Scale score for the undernutrition group (median, 8; interquartile range, 7–8) was not significantly different from that for the normal nutritional status group (median, 8; interquartile range, 7–8). The median rate of increase in a Food Intake Level Scale score was significantly higher (*p* < 0.001) in the undernutrition group (median, 1; interquartile range, 0–5) than in the normal nutrition status group (median, 0; interquartile range, 0–2). The median Barthel Index score at baseline for the undernutrition group (median, 30; interquartile range, 10–50) was not significantly different from that for the normal nutrition status group (median, 25; interquartile range, 5–50). The median Barthel Index score at follow-up was significantly lower (*p* < 0.001) in the undernutrition group (median, 50; interquartile range, 20–70) than in the normal nutrition status group (median, 60; interquartile range, 30–90). The median rate of increase in Barthel Index score was significantly lower (*p* < 0.001) in the undernutrition group (median, 10; interquartile range, 0–28) than in the normal nutrition status group (median, 20; interquartile range, 5–40). Compared with the normal nutrition status group, the undernutrition group had significantly lower increases in the Barthel Index at baseline and at follow-up (*p* < 0.001).

Table 4 displays the results of regression analysis for determining improvements in the Food Intake Level Scale and Barthel Index scores adjusted for age, sex, Charlson Comorbidity Index and prehospitalization facilities. Undernutrition diagnosed using the GLIM criteria was independently associated with the Food Intake Level Scale gain (partial regression coefficient (B) = −0.633, 95% confidence interval (CI) = −1.099 to −0.167) and Barthel Index gain (B = −8.414, 95% CI = −13.089 to −3.739).

## 4. Discussion

This multicenter, retrospective cohort study of patients with dysphagia revealed the following points: First, compared with normal nutritional status, undernutrition diagnosed using the GLIM criteria was associated with poor improvement in swallowing function. Second, undernutrition affected the improvement in activities of daily living. The current study is among the few that examined improvement in swallowing function and activities of daily living in patients with dysphagia and undernutrition diagnosed using the GLIM criteria.

Multivariate analysis revealed that undernutrition negatively affects the gain on the Food Intake Level Scale. Previous studies have shown that undernutrition negatively affects the improvement in swallowing function. Deteriorated nutritional status affects the improvement in dysphagia [26]. Furthermore, it increases the risk of weight loss, low BMI and decreased skeletal muscle mass [27]. Thus, undernutrition leads to a decrease in overall muscle mass (sarcopenia), and swallowing-related muscles, and may result in dysphagia. 

Worse improvement in swallowing function could be attributed to the loss of muscle mass and strength that were assessed by the GLIM criteria. Undernutrition causes dysphagia due to a decrease in total body skeletal muscle mass and muscle strength related to swallowing [1,8]. In this study, compared with the normal nutritional status group, the undernutrition group had a significantly lower body mass index and calf circumference, a measure of muscle mass and handgrip strength and a measure of muscle strength. Grip strength is correlated with tongue muscle strength [28] and low tongue pressure with poor improvement in swallowing function [16]. Furthermore, body mass index and reduced skeletal muscle mass are considered risk factors of dysphagia in older hospitalized adult patients [1,29]. Thus, the patients with undernutrition, who had a lower muscle mass and strength, may have poor improvement in swallowing function. Poor muscle mass and strength in undernourished patients may also attribute to the development of dysphagia in sarcopenia.

Moreover, undernutrition affects improvement in activities of daily living. In rehabilitative patients, undernutrition was associated with poor activities of daily living [27,30]. Undernutrition inhibits improvement in activities of daily living due to muscle weakness, wasting and physical frailty and complications during hospitalization [27]. Another study showed that undernutrition was associated with poor functional outcomes and a higher percentage of weight loss [31]. In this study, undernutrition was associated with a significantly higher percentage of weight loss compared with normal nutritional status. Weight loss can decrease the capability to perform activities of daily living, including physical components such as mobility limitations [32,33]. Therefore, the association of GLIM-criteria-defined undernutrition, including loss of muscle mass, with improvement in activities of daily living seems plausible and supports the findings from a previous study.

Nutritional status and rehabilitation are important in improving the swallowing function and activities of daily living in patients with dysphagia and undernutrition. Much of feeding and swallowing problems affected by sarcopenia may be avoidable [34,35]. Given the pathogenesis mechanism, nutritional management that considers muscle mass and function is critical. Undernutrition and sarcopenia can be accurately assessed to identify subjects at risk of developing the disease. The GLIM criteria are useful for detecting malnutrition. To put it another way, in patients with sarcopenic dysphagia, both nutritional management and physical intervention can improve general muscle strength and function, including swallowing muscles [36]. Furthermore, individualized, high-frequency nutritional support with the multidisciplinary intervention was associated with better nutritional status, physical function and dysphagia in poststroke survivors with undernutrition [37]. These reports showed the importance of improving nutritional status and rehabilitation in patients with dysphagia and undernutrition. 

Rehabilitation nutrition has been shown to improve swallowing function and activities of daily living in patients with dysphagia and malnutrition [38]. Rehabilitation nutrition is a combination of rehabilitation and nutritional therapy. Although there are few clinical studies examining the efficacy of nutritional support for dysphagia in sarcopenia, multidisciplinary feeding and swallowing rehabilitation, as well as nutritional care, may be beneficial in the treatment of sarcopenia dysphagia [38,39,40].

The current study had some limitations. First, the severity of undernutrition diagnosed by the GLIM criteria was not evaluated. Therefore, the degree of improvement in swallowing function and activities of daily living according to undernutrition severity is unknown. Second, this study only included patients with the Food Intake Level Scale ≤8. Therefore, the generalizability of the study results must be examined in patients with dysphagia assessed by gold-standard methods (e.g., videofluorography). Third, research is needed to compare the differences in prevention, treatment and nutritional management between sarcopenia eating and swallowing disorders and other eating and swallowing disorders. Because dysphagia is a complex condition caused by a variety of factors, factors other than nutritional management must be considered. 

## 5. Conclusions

Patients with dysphagia who presented undernutrition diagnosed by the GLIM criteria demonstrated inadequate improvement in swallowing function and ability to perform activities of daily living than those who presented with normal nutritional status. Nevertheless, further studies are needed to examine the effects of rehabilitation nutrition in patients with dysphagia who presented with undernutrition. The significance of this study is that it evaluated the predictive validity of the GLIM criteria, which may help improve activities of daily living in patients with dysphagia.

## Figures and Tables

**Table 1:** Demographic characteristics of the participants at baseline.

	All Patients *n* = 440	Patients with Undernutrition Diagnosed Using the GLIM Criteria *n* = 281	Patients with Normal Nutritional Status *n* = 159	*p*-Value
Age, years, mean ± SD	80.2 ± 10.9	81.8 ± 10.5	77.3 ± 11.0	<0.001 ^(a)^
Sex, female, *n* (%)	224 (50.9)	152 (54.1)	72 (45.3)	0.091 ^(b)^
Prehospitalization facilities, *n* (%)				0.223 ^(c)^
Acute-care hospitals	187 (42.5)	125 (44.5)	62 (39.0)	
Rehabilitation hospitals	209 (47.5)	124 (44.1)	85 (53.5)	
Long-term care hospitals	41 (9.3)	30 (10.7)	11 (6.9)	
Others	3 (0.7)	2 (0.7)	1 (0.6)	
Body mass index (kg/m^2^), mean ± SD	20.2 ± 3.7	19.6 ± 3.5	21.4 ± 3.8	<0.001 ^(a)^
Calf circumference (cm), median (IQR)	28 (25, 31)	27 (25, 30)	30 (26, 33)	<0.001 ^(d)^
Handgrip strength (kg), median (IQR)	12.0 (6.3, 19.0)	11.0 (6.0, 17.5)	14.2 (8.1, 22.1)	<0.001 ^(d)^
C-reactive protein levels (mg/dL), median (IQR)	0.7 (0.2, 2.9)	1.0 (0.3, 5.2)	0.2 (0.1, 0.7)	<0.001 ^(d)^
Serum albumin levels (g/dL), median (IQR)	3.4 (3.0, 3.7)	3.3 (2.9, 3.6)	3.5 (3.3, 3.9)	<0.001 ^(d)^
Charlson Comorbidity Index, median (IQR)	2 (0, 4)	2 (1, 4)	2 (0, 3)	<0.001 ^(d)^

SD, standard deviation; IQR, interquartile range; ^(a)^ Student’s *t*-test; ^(b)^ Chi-square test; ^(c)^ Fisher’s exact test; ^(d)^ Mann–Whitney U test.

**Table 2:** Global Leadership Initiative on Malnutrition criteria.

	Patients with Undernutrition Diagnosed Using the GLIM Criteria *n* = 281	Patients with Normal Nutritional Status *n* = 159	*p*-Value
Phenotypic criteria, presence, *n* (%)			
Weight loss	41 (9.4)	7 (1.6)	<0.001 ^(a)^
Low body mass index	158 (36.0)	53 (12.1)	<0.001 ^(a)^
Reduced muscle mass	276 (63.0)	118 (26.9)	<0.001 ^(a)^
Etiologic criteria, presence, *n* (%)			
Reduced food intake or assimilation	160 (36.4)	8 (1.8)	<0.001 ^(a)^
Disease burden/inflammation	170 (38.7)	13 (3.0)	<0.001 ^(a)^

^(a)^ Chi-square test.

**Table 3:** Univariate analysis of Functional Intake Level Scale score and Barthel Index.

	Patients with Undernutrition Diagnosed Using the GLIM Criteria *n* = 281	Patients with Normal Nutritional Status *n* = 159	*p*-Value
Functional Intake Level Scale score, median (IQR)			
Admission	7 (5, 8)	7 (2, 7)	<0.001 ^(a)^
Follow-up	8 (7, 8)	8 (7, 8)	0.262 ^(a)^
Change	1 (0, 5)	0 (0, 2)	<0.001 ^(a)^
Barthel Index, median (IQR)			
Admission	30 (10, 50)	25 (5, 50)	0.224 ^(a)^
Follow-up	50 (20, 70)	60 (30, 90)	<0.001 ^(a)^
Change	10 (0, 28)	20 (5, 40)	<0.001 ^(a)^

IQR, interquartile range; ^(a)^ Mann–Whitney U test.

**Table 4:** Multiple regression analysis of changes in the Functional Intake Level Scale score and Barthel Index score.

	Change in the Functional Intake Level Scale Score				Change in the Barthel Index Score			
	B	95% Confidence Interval		*p*-Value	B	95% Confidence Interval		*p*-Value
		Lower	Upper			Lower	Upper	
Age	−0.051	−0.072	−0.029	<0.001	−0.201	−0.416	0.014	0.067
Sex	−0.392	−0.849	0.064	0.092	−0.277	−4.857	4.303	0.905
Charlson Comorbidity Index	−0.098	−0.220	0.024	0.116	−1.859	−3.085	−0.633	<0.003
Prehospitalization facilities	−1.159	−1.497	−0.821	<0.001	−0.547	−3.938	2.844	0.751
Undernutrition diagnosed using the GLIM criteria	−0.633	−1.099	−0.167	<0.008	−8.414	−13.089	−3.739	<0.001

B, partial regression coefficient; GLIM, Global Leadership Initiative on Malnutrition.

## Data Availability

Not applicable.
